# Supplementary material for: Iterative improvement in the automatic modular design of robot swarms
Source: PeerJ Comput Sci. 2020 Dec 7;6:e322. doi: 10.7717/peerj-cs.322 (PMC7924708; doi:10.7717/peerj-cs.322)
Supplement: Supplemental Information 3 [file peerj-cs-06-322-s003.zip › argos3/doc/api/standalone/a00588_source.html]

ARGoS: plugins/simulator/entities/rab\_equipped\_entity.cpp Source File


- Main Page
- Related Pages
- Namespaces
- Classes
- Files

- File List
- File Members

# plugins/simulator/entities/rab\_equipped\_entity.cpp

Go to the documentation of this file.

```
00001 
00007 #include "rab_equipped_entity.h"
00008 #include <argos3/core/utility/string_utilities.h>
00009 #include <argos3/core/simulator/simulator.h>
00010 #include <argos3/core/simulator/space/space.h>
00011 #include <argos3/core/simulator/entity/composable_entity.h>
00012 #include <argos3/core/simulator/entity/embodied_entity.h>
00013 
00014 namespace argos {
00015 
00016    /****************************************/
00017    /****************************************/
00018 
00019    CRABEquippedEntity::CRABEquippedEntity(CComposableEntity* pc_parent) :
00020       CPositionalEntity(pc_parent),
00021       m_psAnchor(NULL),
00022       m_fRange(0.0f),
00023       m_pcEntityBody(NULL) {
00024       Disable();
00025    }
00026 
00027    /****************************************/
00028    /****************************************/
00029 
00030    CRABEquippedEntity::CRABEquippedEntity(CComposableEntity* pc_parent,
00031                                           const std::string& str_id,
00032                                           size_t un_msg_size,
00033                                           Real f_range,
00034                                           SAnchor& s_anchor,
00035                                           CEmbodiedEntity& c_entity_body,
00036                                           const CVector3& c_pos_offset,
00037                                           const CQuaternion& c_rot_offset) :
00038       CPositionalEntity(pc_parent,
00039                         str_id),
00040       m_psAnchor(&s_anchor),
00041       m_cPosOffset(c_pos_offset),
00042       m_cRotOffset(c_rot_offset),
00043       m_cData(un_msg_size),
00044       m_fRange(f_range),
00045       m_pcEntityBody(&c_entity_body) {
00046       Disable();
00047       CVector3 cPos = c_pos_offset;
00048       cPos.Rotate(s_anchor.Orientation);
00049       cPos += s_anchor.Position;
00050       SetInitPosition(cPos);
00051       SetPosition(cPos);
00052       SetInitOrientation(s_anchor.Orientation * c_rot_offset);
00053       SetOrientation(GetInitOrientation());
00054    }
00055 
00056    /****************************************/
00057    /****************************************/
00058 
00059    void CRABEquippedEntity::Init(TConfigurationNode& t_tree) {
00060       try {
00061          /*
00062           * Init entity.
00063           * Here we explicitly avoid to call CPositionalEntity::Init() because that
00064           * would also initialize position and orientation, which, instead, must
00065           * be calculated from reference entity and offsets.
00066           */
00067          CEntity::Init(t_tree);
00068          /* Get offsets */
00069          GetNodeAttributeOrDefault(t_tree, "pos_offset", m_cPosOffset, m_cPosOffset);
00070          std::string strRotOffset;
00071          GetNodeAttributeOrDefault(t_tree, "rot_offset", strRotOffset, strRotOffset);
00072          if(strRotOffset != "") {
00073             CDegrees cRotOffsetEuler[3];
00074             ParseValues(strRotOffset, 3, cRotOffsetEuler, ',');
00075             m_cRotOffset.FromEulerAngles(ToRadians(cRotOffsetEuler[0]),
00076                                          ToRadians(cRotOffsetEuler[1]),
00077                                          ToRadians(cRotOffsetEuler[2]));
00078          }
00079          /* Parse and look up the anchor */
00080          std::string strAnchorId;
00081          GetNodeAttribute(t_tree, "anchor", strAnchorId);
00082          /*
00083           * NOTE: here we get a reference to the embodied entity
00084           * This line works under the assumption that:
00085           * 1. the RABEquippedEntity has a parent;
00086           * 2. the parent has a child whose id is "body"
00087           * 3. the "body" is an embodied entity
00088           * If any of the above is false, this line will bomb out.
00089           */
00090          m_pcEntityBody = &GetParent().GetComponent<CEmbodiedEntity>("body");
00091          m_psAnchor = &m_pcEntityBody->GetAnchor(strAnchorId);
00092          /* Get message size */
00093          size_t unMsgSize;
00094          GetNodeAttribute(t_tree, "msg_size", unMsgSize);
00095          m_cData.Resize(unMsgSize);
00096          /* Get transmission range */
00097          GetNodeAttribute(t_tree, "range", m_fRange);
00098          /* Set init position and orientation */
00099          Update();
00100          SetInitPosition(GetPosition());
00101          SetInitOrientation(GetOrientation());
00102       }
00103       catch(CARGoSException& ex) {
00104          THROW_ARGOSEXCEPTION_NESTED("Error initializing a range and bearing entity \"" << GetId() << "\"", ex);
00105       }
00106    }
00107 
00108    /****************************************/
00109    /****************************************/
00110 
00111    void CRABEquippedEntity::Update() {
00112       CVector3 cPos = m_cPosOffset;
00113       cPos.Rotate(m_psAnchor->Orientation);
00114       cPos += m_psAnchor->Position;
00115       SetPosition(cPos);
00116       SetOrientation(m_psAnchor->Orientation * m_cRotOffset);
00117    }
00118 
00119    /****************************************/
00120    /****************************************/
00121 
00122    void CRABEquippedEntity::Reset() {
00123       m_cData.Zero();
00124    }
00125 
00126    /****************************************/
00127    /****************************************/
00128 
00129    void CRABEquippedEntity::Enable() {
00130       if(m_psAnchor) m_psAnchor->Enable();
00131    }
00132 
00133    /****************************************/
00134    /****************************************/
00135 
00136    void CRABEquippedEntity::Disable() {
00137       if(m_psAnchor) m_psAnchor->Disable();
00138    }
00139 
00140    /****************************************/
00141    /****************************************/
00142 
00143    void CRABEquippedEntity::SetData(const CByteArray& c_data) {
00144       if(m_cData.Size() == c_data.Size()) {
00145          m_cData = c_data;
00146       }
00147       else {
00148          THROW_ARGOSEXCEPTION("CRABEquippedEntity::SetData() : data size does not match, expected " << m_cData.Size() << ", got " << c_data.Size());
00149       }
00150    }
00151 
00152    /****************************************/
00153    /****************************************/
00154 
00155    void CRABEquippedEntity::ClearData() {
00156       m_cData.Zero();
00157    }
00158 
00159    /****************************************/
00160    /****************************************/
00161 
00162    void CRABEquippedEntitySpaceHashUpdater::operator()(CAbstractSpaceHash<CRABEquippedEntity>& c_space_hash,
00163                                                        CRABEquippedEntity& c_element) {
00164       /* Calculate the position of the center of the RAB equipped entity in the space hash */
00165       c_space_hash.SpaceToHashTable(m_nCenterI,
00166                                     m_nCenterJ,
00167                                     m_nCenterK,
00168                                     c_element.GetPosition());
00169       /* Update the cells in a sphere around it */
00170       SInt32 nRangeI = c_space_hash.SpaceToHashTable(c_element.GetRange(), 0);
00171       SInt32 nRangeJ;
00172       SInt32 nRangeK;
00173       for(SInt32 i = 0; i <= nRangeI; ++i) {
00174          nRangeJ =
00175             c_space_hash.SpaceToHashTable(
00176                ::sqrt(
00177                   Square(c_element.GetRange()) -
00178                   Square(c_space_hash.HashTableToSpace(i, 0))
00179                   ),
00180                1);
00181          for(SInt32 j = 0; j <= nRangeJ; ++j) {
00182             nRangeK =
00183                c_space_hash.SpaceToHashTable(
00184                   ::sqrt(
00185                      Square(c_element.GetRange()) -
00186                      Square(c_space_hash.HashTableToSpace(j, 1))
00187                      ),
00188                   2);
00189             for(SInt32 k = 0; k <= nRangeK; ++k) {
00190                if(i > 0) {
00191                   /*
00192                    * i > 0
00193                    */
00194                   if(j > 0) {
00195                      /*
00196                       * i > 0
00197                       * j > 0
00198                       */
00199                      if(k > 0) {
00200                         /*
00201                          * i > 0
00202                          * j > 0
00203                          * k > 0
00204                          */
00205                         c_space_hash.UpdateCell(m_nCenterI + i, m_nCenterJ + j, m_nCenterK + k, c_element);
00206                         c_space_hash.UpdateCell(m_nCenterI + i, m_nCenterJ + j, m_nCenterK - k, c_element);
00207                         c_space_hash.UpdateCell(m_nCenterI + i, m_nCenterJ - j, m_nCenterK + k, c_element);
00208                         c_space_hash.UpdateCell(m_nCenterI + i, m_nCenterJ - j, m_nCenterK - k, c_element);
00209                         c_space_hash.UpdateCell(m_nCenterI - i, m_nCenterJ + j, m_nCenterK + k, c_element);
00210                         c_space_hash.UpdateCell(m_nCenterI - i, m_nCenterJ + j, m_nCenterK - k, c_element);
00211                         c_space_hash.UpdateCell(m_nCenterI - i, m_nCenterJ - j, m_nCenterK + k, c_element);
00212                         c_space_hash.UpdateCell(m_nCenterI - i, m_nCenterJ - j, m_nCenterK - k, c_element);
00213                      }
00214                      else {
00215                         /*
00216                          * i > 0
00217                          * j > 0
00218                          * k == 0
00219                          */
00220                         c_space_hash.UpdateCell(m_nCenterI + i, m_nCenterJ + j, m_nCenterK, c_element);
00221                         c_space_hash.UpdateCell(m_nCenterI + i, m_nCenterJ - j, m_nCenterK, c_element);
00222                         c_space_hash.UpdateCell(m_nCenterI - i, m_nCenterJ + j, m_nCenterK, c_element);
00223                         c_space_hash.UpdateCell(m_nCenterI - i, m_nCenterJ - j, m_nCenterK, c_element);
00224                      }
00225                   }
00226                   else {
00227                      /*
00228                       * i > 0
00229                       * j == 0
00230                       */
00231                      if(k > 0) {
00232                         /*
00233                          * i > 0
00234                          * j == 0
00235                          * k > 0
00236                          */
00237                         c_space_hash.UpdateCell(m_nCenterI + i, m_nCenterJ, m_nCenterK + k, c_element);
00238                         c_space_hash.UpdateCell(m_nCenterI + i, m_nCenterJ, m_nCenterK - k, c_element);
00239                         c_space_hash.UpdateCell(m_nCenterI - i, m_nCenterJ, m_nCenterK + k, c_element);
00240                         c_space_hash.UpdateCell(m_nCenterI - i, m_nCenterJ, m_nCenterK - k, c_element);
00241                      }
00242                      else {
00243                         /*
00244                          * i > 0
00245                          * j == 0
00246                          * k == 0
00247                          */
00248                         c_space_hash.UpdateCell(m_nCenterI + i, m_nCenterJ, m_nCenterK, c_element);
00249                         c_space_hash.UpdateCell(m_nCenterI - i, m_nCenterJ, m_nCenterK, c_element);
00250                      }
00251                   }
00252                }
00253                else {
00254                   /*
00255                    * i == 0
00256                    */
00257                   if(j > 0) {
00258                      /*
00259                       * i == 0
00260                       * j > 0
00261                       */
00262                      if(k > 0) {
00263                         /*
00264                          * i == 0
00265                          * j > 0
00266                          * k > 0
00267                          */
00268                         c_space_hash.UpdateCell(m_nCenterI, m_nCenterJ + j, m_nCenterK + k, c_element);
00269                         c_space_hash.UpdateCell(m_nCenterI, m_nCenterJ + j, m_nCenterK - k, c_element);
00270                         c_space_hash.UpdateCell(m_nCenterI, m_nCenterJ - j, m_nCenterK + k, c_element);
00271                         c_space_hash.UpdateCell(m_nCenterI, m_nCenterJ - j, m_nCenterK - k, c_element);
00272                      }
00273                      else {
00274                         /*
00275                          * i == 0
00276                          * j > 0
00277                          * k == 0
00278                          */
00279                         c_space_hash.UpdateCell(m_nCenterI, m_nCenterJ + j, m_nCenterK, c_element);
00280                         c_space_hash.UpdateCell(m_nCenterI, m_nCenterJ - j, m_nCenterK, c_element);
00281                      }
00282                   }
00283                   else {                     
00284                      /*
00285                       * i == 0
00286                       * j == 0
00287                       */
00288                      if(k > 0) {
00289                         /*
00290                          * i == 0
00291                          * j == 0
00292                          * k > 0
00293                          */
00294                         c_space_hash.UpdateCell(m_nCenterI, m_nCenterJ, m_nCenterK + k, c_element);
00295                         c_space_hash.UpdateCell(m_nCenterI, m_nCenterJ, m_nCenterK - k, c_element);
00296                      }
00297                      else {
00298                         /*
00299                          * i == 0
00300                          * j == 0
00301                          * k == 0
00302                          */
00303                         c_space_hash.UpdateCell(m_nCenterI, m_nCenterJ, m_nCenterK, c_element);
00304                      }
00305                   }
00306                }
00307             }
00308          }
00309       }
00310    }
00311 
00312    /****************************************/
00313    /****************************************/
00314 
00315    REGISTER_STANDARD_SPACE_OPERATIONS_ON_ENTITY(CRABEquippedEntity);
00316 
00317    /****************************************/
00318    /****************************************/
00319 
00320    CRABEquippedEntityGridCellUpdater::CRABEquippedEntityGridCellUpdater(CGrid<CRABEquippedEntity>& c_grid) :
00321       m_cGrid(c_grid) {}
00322    
00323    bool CRABEquippedEntityGridCellUpdater::operator()(SInt32 n_i,
00324                                                       SInt32 n_j,
00325                                                       SInt32 n_k,
00326                                                       CGrid<CRABEquippedEntity>::SCell& s_cell) {
00327       /* Update cell */
00328       m_cGrid.UpdateCell(n_i, n_j, n_k, *m_pcEntity);
00329       /* Continue with other cells */
00330       return true;
00331    }
00332    
00333    void CRABEquippedEntityGridCellUpdater::SetEntity(CRABEquippedEntity& c_entity) {
00334       m_pcEntity = &c_entity;
00335    }
00336 
00337    CRABEquippedEntityGridEntityUpdater::CRABEquippedEntityGridEntityUpdater(CGrid<CRABEquippedEntity>& c_grid) :
00338       m_cGrid(c_grid),
00339       m_cCellUpdater(c_grid) {}
00340 
00341    bool CRABEquippedEntityGridEntityUpdater::operator()(CRABEquippedEntity& c_entity) {
00342       try {
00343          m_cCellUpdater.SetEntity(c_entity);
00344          m_cGrid.ForCellsInBoxRange(c_entity.GetPosition(),
00345                                     CVector3(c_entity.GetRange(),
00346                                              c_entity.GetRange(),
00347                                              c_entity.GetRange()),
00348                                     m_cCellUpdater);
00349          /* Continue with the other entities */
00350          return true;
00351       }
00352       catch(CARGoSException& ex) {
00353          THROW_ARGOSEXCEPTION_NESTED("While updating the RAB entity grid for RAB entity \"" << c_entity.GetContext() << c_entity.GetId() << "\"", ex);
00354       }
00355    }
00356 
00357    /****************************************/
00358    /****************************************/
00359 
00360 }
```

---

Generated on 10 Jul 2018 for ARGoS by 
 1.6.1
